# Architecture for Directed Transport of Superparamagnetic Microbeads in a Magnetic Domain Wall Routing Network

**DOI:** 10.1038/s41598-017-10149-9

**Published:** 2017-08-31

**Authors:** Elizabeth Rapoport, Geoffrey S. D. Beach

**Affiliations:** Department of Materials Science and Engineering, Massachusetts Institute of Technology, Cambridge, Massachusetts, 02139 USA

## Abstract

Directed transport of biological species across the surface of a substrate is essential for realizing lab-on-chip technologies. Approaches that utilize localized magnetic fields to manipulate magnetic particles carrying biological entities are attractive owing to their sensitivity, selectivity, and minimally disruptive impact on biomaterials. Magnetic domain walls in magnetic tracks produce strong localized fields and can be used to capture, transport, and detect individual superparamagnetic microbeads. The dynamics of magnetic microbead transport by domain walls has been well studied. However, demonstration of more complex functions such as selective motion and sorting using continuously driven domain walls in contiguous magnetic tracks is lacking. Here, a junction architecture is introduced that allows for branching networks in which superparamagnetic microbeads can be routed along dynamically-selected paths by a combination of rotating in-plane field for translation, and a pulsed out-of-plane field for path selection. Moreover, experiments and modeling show that the select-field amplitude is bead-size dependent, which allows for digital sorting of multiple bead populations using automated field sequences. This work provides a simple means to implement complex routing networks and selective transport functionalities in chip-based devices using magnetic domain wall conduits.

## Introduction

Recently, there has been considerable interest to develop faster, cheaper, and more sensitive devices for medical diagnostics and biomedical research. These so-called “point-of-care” or “lab-on-chip” technologies promise to bring diagnostics closer to the patient, reduce sample volume requirements, and scale down device size while simultaneously increasing functionality and precision. Microfluidic approaches^1–5^ are conventionally used, and detection capabilities in such systems are usually based on optical^6–8^ or electrical^9–11^ transducers using micro- or nanoparticles. Although much progress has been made, microfluidic systems still require carefully-engineered channels and off-chip accessories, and optical and electrical sensing and actuation are highly sensitive to properties and variations in the fluid matrix. In this work, we demonstrate a magnetic architecture that can be used to design a complete and dynamically controlled routing network that can be readily integrated with a simple magneto-mechanical detection mechanism^11, 12^.

Magnetic systems are particularly attractive for a variety of reasons. Intricate channeling is not required. Magnetic beads can be manipulated by fields, representing an extra degree of freedom over non-magnetic beads and allowing “action at a distance”^13^. Magnetic forces can be localized, even to the level of single beads^11, 12, 14–24^. Magnetic signals cannot be quenched and magnetic fields do not interfere with biological processes. And a high level of selectivity can be achieved, due to the difference in susceptibility between magnetic beads and non-magnetic samples.

Because of their many advantages, magnetic technologies for bead manipulation have steadily progressed. Microscale electromagnets^25–28^ and two-dimensional arrays of soft magnetic microstructures^14, 16, 29–32^ have been used to transport microbead ensembles^26, 29, 30^ and even individual beads^14, 16, 31, 32^. Extended magnetic track structures have also been introduced, which allow for one-dimensional transport and the construction of multiplexed networks^11, 15, 17–23, 33–37^. These transport systems are based on periodic, nonuniform magnetic textures or propagating magnetic domain walls (DWs) that lead to either stepped or continuously translating potential energy wells that can transport individual magnetic particles. The strong, localized stray field^38^ from domain walls in submicrometer tracks can trap individual superparamagnetic (SPM) beads with forces up to hundreds of pN^15, 18, 22, 23, 33, 39^. As DWs can be readily driven along a track by a magnetic field,^40–43^ spin-polarized electric current^44, 45^, or electric fields^46^ they can serve as mobile magnetic traps for single bead transport along a predefined path. Indeed, Vieira *et al*.^15^ demonstrated that DWs in zig-zag magnetic nanotracks can be used to capture and release SPM microbeads and magnetically tagged entities and shuttle them across the surface of a chip. Donolato *et al*.^18^ extended this work to show that not only could beads follow a traveling DW potential, but that they could precisely track it in a curved structure. Designs in which a DW is continuously translated while continuously binding a magnetic particle have some advantages compared to systems in which beads jump stepwise from one localized stray field source to another: higher transport speeds can be obtained by eliminating the diffusive transport step between binding sites, and particles can be more robust in, e.g., fluid flows since the particles are always magnetically bound during transport. Recently, a curved track architecture was introduced in which a rotating magnetic field can continuously drive magnetic domain walls along a track at a speed set by the field rotation rate, and that very high particle transport speeds in excess of 1000 μm/s can be obtained^22, 23^.

**Figure 1:**
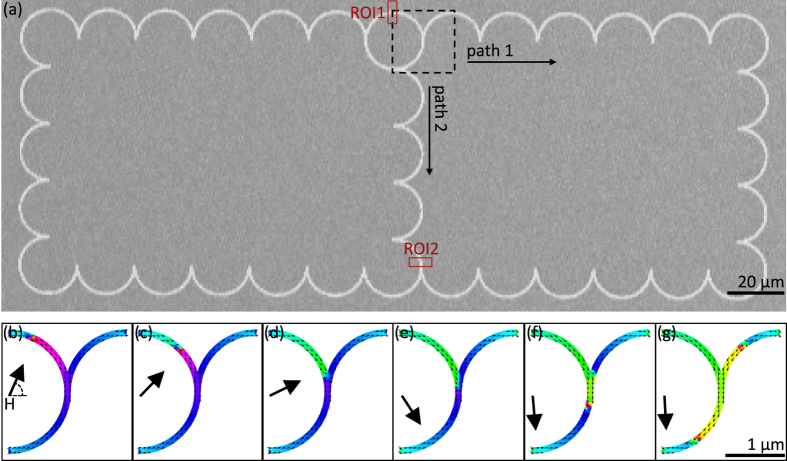
Magnetic track and simulations of domain wall motion. (**a**) Optical image of closed-loop branched curvilinear Ni_80_Fe_20_ track composed of 20 $\mu$m outer diameter, 800 nm wide, 40 nm thick linked semi-circular segments. Dashed square indicates micromagnetically simulated junction region. Rectangles marked ROI1 and ROI2 represent regions of interest used for particle tracking during experiments (see text). Path 1 and Path 2 represent two possible routes for bead travel past the junction. (**b–g**) Snapshots of micromagnetically-simulated magnetization configuration in junction region in the presence of a rotating magnetic field. The region corresponds to a 2 $\mu$m outer diameter, 100 nm wide, 60 nm thick Ni_80_Fe_20_ junction as an externally applied in-plane field (arrow) is rotated in time. A single head-to-head domain wall enters the junction, and two domain walls, one head-to-head and one tail-to-tail, exit.

In this work, we show that the bead-DW system is indeed a viable candidate for such an architecture, and add an essential routing capability to the set of previously demonstrated DW-mediated bead handling functions e.g. capture, transport, detection, and release^11, 12, 22, 23^. Using a curvilinear backbone, in which we have the fine control over DW position and speed^23^ necessary for bead handling, we show that the direction of bead motion at junctions in branched structures (Fig. 1(a)) can be precisely selected by application of a vertical field. Numerical work is presented in support of the theoretical basis for selective motion, and experiment reveals a threshold vertical select-field for a sample of nominally identical beads. With these results, we show the reproducibility of this DW-driven bead routing technique, and the ability to sort a mixed population of SPM beads by simple application of a vertical field, thereby advancing the realization of an integrated magnetic lab-on-chip device.

## Simulation

### Domain wall motion through a curvilinear junction

In order to investigate the behavior of a bead at a junction in a branched curvilinear structure, as in Fig. 1(a), the motion of a DW through a junction (Fig. 1(a), black square) was first calculated micromagnetically using the Object-Oriented MicroMagnetic Framework (OOMMF) platform. A vortex DW was initialized in a model track junction 100 nm wide, 60 nm thick, and with a 2 μm outer diameter. The DW-containing junction was then subjected to a rotating field of 625 Oe. In each simulation stage, the field angle was stepped clockwise 2 degrees, and the spin configuration in the strip was allowed to relax for the duration of the stage (5 ns). The simulation assumed materials parameters for bulk Ni_80_Fe_20_ (exchange constant $A=$ 1.3$\times$10^–11^ J m^−1^, saturation magnetization ${M}_{s}=$800 kA m^−1^, uniaxial anisotropy ${K}_{u}=$0 J m^3^), and used a cell size of 2.5 $\times$ 2.5 $\times$ 60 nm^3^ and a damping parameter *α* = 1. The spin configuration in the junction as a function of rotating in-plane field is shown in Fig. 1(b–g). The initialized DW is held pinned at the inlet of the structure with an in-plane field (Fig. 1(b)). As the field is rotated in-plane, the DW begins to move in the direction of field rotation, tracking the field axis (Fig. 1(c)). Upon entering the junction branch point, the DW becomes attracted and pinned to the local magnetostatic potential well created by the junction notch (Fig. 1(d)). While pinned, the DW does not track with continued field rotation, but rather stretches (Fig. 1(e)) and eventually splits (Fig. 1(f)) into two DWs of opposite configuration i.e. head-to-head (H-H) and tail-to-tail (T-T). The resulting DWs, which now lag behind the field axis, then accelerate around the track to align with the field (Fig. 1(g)).

**Figure 2:**
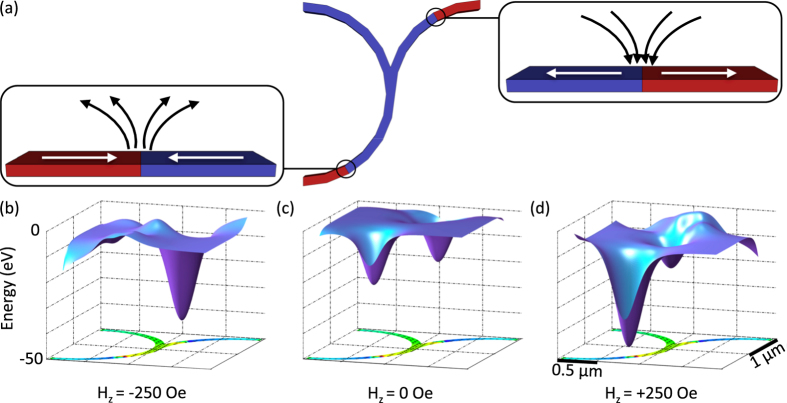
Magnetostatic potential wells near domain wall junction. (**a**) Schematic of resulting domain wall configurations and associated stray fields after propagation of a single head-to-head domain wall through a curvilinear junction. Head-to-head (bottom left) and tail-to-tail (upper right) domain walls yield positively and negatively oriented stray fields, respectively. (**b–d**) Calculated magnetostatic potential energy surfaces for a 300 nm diameter bead over a 2 $\mu$m outer diameter, 100 nm wide, 60 nm thick Ni_80_Fe_20_ junction containing two opposite domain walls, as in **(a)**, in **(b)** −250 Oe, **(c)** 0 Oe, and **(d)** +250 Oe vertical field.

The single incoming DW splitting into two of opposite configuration creates an asymmetry in the system that can be exploited for selective bead motion. Here, both of the DWs of opposite configuration exiting the junction can act as magnetostatic potential energy wells for a bead, yielding two possible paths for bead motion. However, the stray field above the two DWs is of opposite sign (Fig. 2(a)), and thus an externally applied vertical field can be used to strengthen the bead-DW interaction for one DW configuration, while simultaneously weakening the strength of interaction for the other, resulting in preferential binding and motion of the bead with one of the two DWs.

We note that since the incoming DW is momentarily pinned before splitting into two DWs (Fig. 1(e–g)), it is likely that the bead is temporarily displaced from the potential minimum of the carrying DW as the DW breaks free from the junction and advances rapidly to realign with the field. This could limit the maximum field rotation rate, so that the bead can diffuse back to the DW potential minimum before the DW advances too far. We note that due to the broad spatial extent of the stray field interaction, the bead is expected to remain bound to the DW even during this transient period, and moreover, since the depinning field at a geometrical junction or constriction scales inversely with the track width^47^, we expect that this effect is much smaller in the experimental system as compared to the simulated structures in Fig. 1. Indeed, in the experiments described below, we do not see evidence for beads being “dropped” at the junction.

### Asymmetric bead interaction with domain walls of opposite configuration under vertical field

The effect of vertical field on bead interaction with the oppositely configured DWs that exit a junction was investigated numerically. The track magnetization profile from a simulation stage at which two DWs are present in the junction was used to compute the stray field above the track via the scalar potential. From the stray field, the magnetostatic potential energy of a spherical SPM bead was estimated by integrating the dipolar energy density $-M\bullet B$ over the bead volume, assuming a bead magnetization $M=\chi B/{\mu }_{0}$ with $\chi /{\mu }_{0}=$ 800 kA m^−1^ T^−1^ from ref. [48] and a sphere demagnetization factor of 1/3. Although it is expected that the presence of the bead may perturb the DW structure, these effects were neglected in the current calculations since prior work has shown them to be negligible^39^.

Figure 2(b–d) show magnetostatic potential energy surfaces for a 300 nm diameter bead over a junction containing two DWs as a function of vertically applied field ${H}_{z}$. For ${H}_{z}=$0 Oe (Fig. 2(c)), the potential energy wells above the two DWs are nominally identical (excluding the contribution from the junction notch). However, with the application of *H*_*z*_ < 0, which is simultaneously additive to the negative stray field above the T-T DW and subtractive to the positive field above the H-H DW, the interaction between the bead and T-T DW is selectively strengthened over that of the bead with the H-H DW (Fig. 2(b)). In the same manner, *H*_*z*_ > 0 is used to preferentially select for a H-H DW over a T-T DW (Fig. 2(d)). In fact, in a strong enough vertical field, a well can be inverted, the beginning stages of which can be seen above the T-T DW in Fig. 2(d). These results show that a vertical field of appropriate sign can be used to impose bead preference for one DW over another. This is the basis for the selection of bead motion at a junction.

## Experiments

### Track design, bead capture, and optical tracking

To verify the response of a bead at a junction to a vertical field, closed-loop branched curvilinear test structures (Fig. 1(a)) were fabricated (see Methods). In such structures, DW-mediated bead transport can be achieved with rotating in-plane fields while simultaneously keeping beads in closed circuits, which is useful for repeat measurements. Experiments used a custom magnet and microscope system described in detail in ref. [11]. During experiments, a dilute suspension of commercial 2.8 μm diameter superparamagnetic beads (see Methods) was placed in a polydimethylsiloxane (PDMS) well on the wafer surface and sealed with a microscope cover slip. A large in-plane drive field applied along the diagonal direction of the track structure (Fig. 1(a)) was then used to initialize DWs within the curvilinear track. The field amplitude (~500 Oe) exceeds the threshold needed to realize the “onion” state in magnetic ring structures and hence ensures DWs in each curved segment^22^. Bead capture by DW fringing fields was monitored via a CCD camera fitted to a custom microscope apparatus and integrated with a custom LabVIEW imaging program allowing region of interest (ROI) definition. Beads within ~10 $\mu$m of the track were subsequently abruptly drawn towards and trapped by the nearest DW. Once a bead was trapped by a DW, a rotating in-plane drive field of ~250 Oe was used to move bead-DW pairs around the track at the drive field frequency.

The motion of individual trapped beads was tracked using the LabVIEW-CCD-microscope assembly. By continuously integrating over ROI pixel greyscale values, bead passage through ROIs was monitored in real time. Using this feature, an ROI (ROI1 in Fig. 1(a)) was defined at the inlet of a junction and used to trigger the application of a vertical field of a specific magnitude, polarity, and duration. The subsequent motion of a bead along either Path 1 or Path 2 (Fig. 1(a)) was then obtained by the absence or passage, respectively, of the bead at a second ROI (ROI2 in Fig. 1(a)). A third ROI was also used along Path 1 to verify that a negative detection at ROI2 corresponded to bead travel along path 1 as opposed to the bead being lost from the track.

### Bead motion at a junction

**Figure 3:**
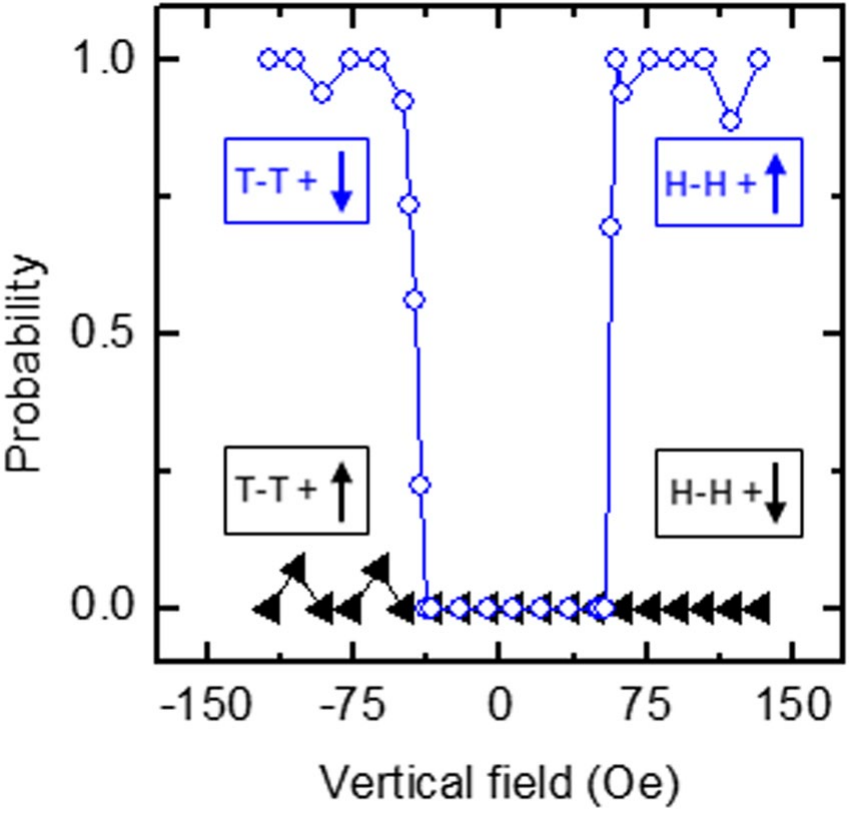
Experimental path selection probabilities. Probability of a M-270 bead taking Path 2 as a function of vertical field polarity and magnitude, and the configuration of the domain wall exiting into Path 2. The two curves represent Path 2 domain wall and field polarity combinations in which the applied field is (filled triangle) subtractive and (open circle) additive to the domain wall stray field.

Repeated measurements were collected of the motion of a 2.8 μm bead across a junction, subject to vertical fields in the range of −150 Oe $< {H}_{z} <$ 150 Oe. Figure 3 shows the probability of the bead taking Path 2 as a function of vertical field polarity and magnitude, and the configuration of the DW (Path 2 DW) exiting into Path 2. The filled triangle data represent combinations of Path 2 DW and vertical field in which the applied field is subtractive to the DW stray field i.e. positive (up) with T-T DWs, or negative (down) with H-H DWs. From simulation results (Fig. 2(b–d)), we expect that the bead-DW interaction is weakened for the Path 2 DW under these conditions and that consequently at no field magnitude should the bead prefer to move along Path 2. This is indeed seen experimentally in the data.

The open circle data represent combinations of Path 2 DW and vertical field in which the applied field is additive to the DW stray field i.e. negative (down) with T-T DWs, or positive (up) with H-H DWs. Under these conditions, the bead is expected to travel along Path 2. These data show a clear threshold vertical select-field value at ~57 Oe, below which the bead travels along Path 1, but above which the preference for the Path 2 DW dominates, and the bead travels along Path 2. From these data it is clear that to achieve directed bead motion at a junction, not only must the vertical field be of appropriate sign, but also of appropriate magnitude. We speculate that the preference for Path 1 in the absence of a vertical field is due to the asymmetry in the DW depinning process at the junction, however, a detailed understanding would likely require simulations using dimensions closer to the experimental values and is beyond the present scope.

It should be noted that the track for Path 2 itself contains a junction identical to the one under investigation. In order to insure a bead did not take the effective Path 2 of Path 2, triggered vertical fields were applied only for the duration of bead passage through the junction of interest. *H*_*z*_ = 0 Oe during bead passage through the junction within Path 2 ensured that the bead continued along Path 2 and back into the circuit.

### Sorting a two-bead population

That under small vertical fields (below the threshold shown in Fig. 3) the bead still travels with the Path 1 DW, despite the enhancement to the interaction with the Path 2 DW, suggests that there is threshold interaction between the bead and Path 2 DW necessary for Path 2 travel. Given that the extent of bead-DW interaction depends on the size and susceptibility of a bead, we investigated whether the observed thresholding in conjunction with differing bead characteristics could be used to realize bead-specific behavior under the same field conditions.

**Figure 4:**
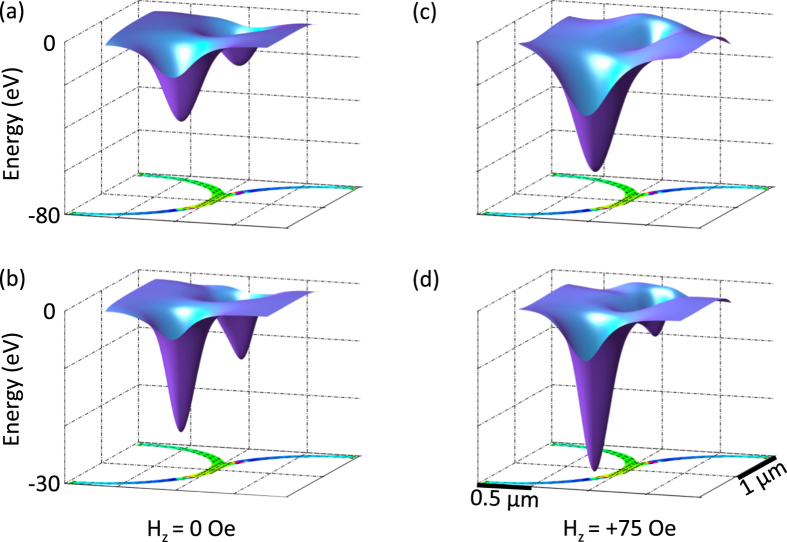
Bead size dependence of potential energy wells at junction. Calculated magnetostatic potential energy surfaces for **(a)** a 600 nm diameter and **(b)** 300 nm diameter bead in 0 Oe vertical field, and **(c)** a 600 nm diameter and **(d)** 300 nm diameter bead in +75 Oe vertical field over a 2 *μ*m outer diameter, 100 nm wide, 60 nm thick Ni_80_Fe_20_ junction containing two oppositely configured domain walls.

The effect of bead size was numerically simulated, and Fig. 4 shows the potential energy surfaces for two beads of different size over a junction under different vertical field conditions. Figure 4(a,b) show energy surfaces over a junction containing two exiting DWs for a 600 nm and 300 nm diameter bead, respectively, in ${H}_{z}=$ 0 Oe. Although the energy landscapes for the two beads exhibit similar overall features, differences arise due to the size discrepancy between the beads. As such, it is expected that the surfaces under ${H}_{z}\ne$ 0 Oe should not respond identically, but rather also be a function of bead size. Indeed, when ${H}_{z}=$ 75 Oe is applied to the 600 nm and 300 nm diameter beads (Fig. 4(c,d)), in both cases the well above the Path 2 DW deepens while that above the Path 1 DW shallows. However, these changes for the two beads are not equivalent. From these results it is expected that the threshold for bead travel with the Path 2 DW is a function of bead properties that affect bead-DW interaction, such as size and susceptibility, and therefor will occur at different values of ${H}_{z}$ for different beads.

**Figure 5:**
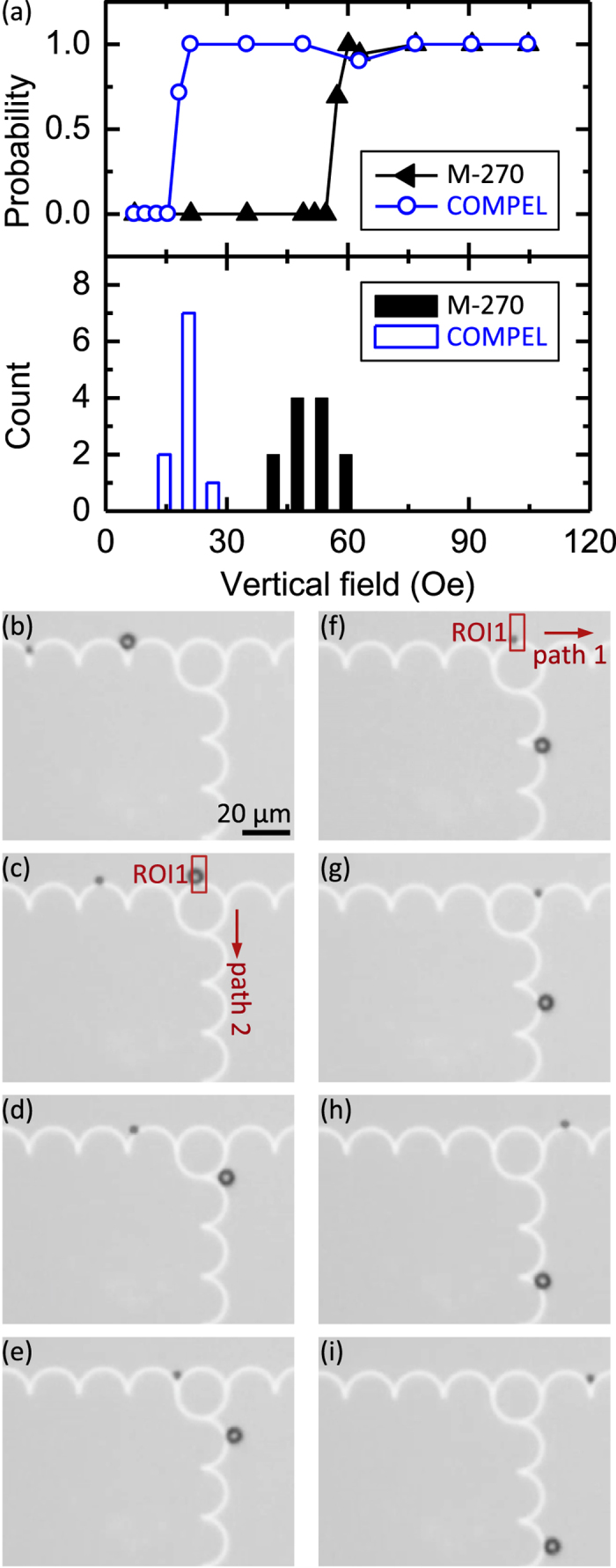
Experimental demonstration of size-selective directed transport. (**a**) Threshold vertical switch-field data for single (top) and population of (bottom) COMPEL and M-270 bead(s). **(b-i)** Differing motion of a COMPEL and M-270 bead through a junction as a 35 Oe vertical field is triggered by bead passage through ROI1 (rectangle) in **(c)** and **(f)**.

The predictions of Fig. 4 were investigated experimentally. Following the approach described above for the collection of Fig. 3 data, repeated measurements of the motion across a junction of two beads from two different bead populations were collected. Here, we used a mixture of commercial monodisperse 2.8 μm (small) and 5.8 μm (large) commercial beads (see methods). In these measurements, the polarity of ${H}_{z}$ was programmed to always enhance the bead interaction with the Path 2 DW, regardless of the incoming DW configuration. The probability for each bead taking Path 2 was then calculated as a function of vertical field magnitude and plotted in Fig. 5(a, top). The open circle curve represents data for a 5.8 μm diameter superparamagnetic bead, and the filled triangle curve represents data for a 2.8 μm bead. There is a clear shift in threshold vertical select-field, corroborating the predictions of Fig. 4. To test the reproducibility of these results, Path 2 probability versus ${H}_{z}$ curves were obtained for 10 large beads and 12 small beads. A MATLAB fitting program was then used to extract the threshold vertical select-field from each curve. This point was taken to be the ${H}_{z}$ at which the probability of the bead taking Path 2 was 0.5. The results of these statistics are plotted in Fig. 5(a, bottom), with the open and filled bars representing the vertical select-field for large and small beads, respectively. Again, there is a significant shift in the threshold vertical select-field between the two populations, with the select-field for the large and small beads having a mean of 19 Oe and 52 Oe, respectively.

Given these results, it follows that a vertical field of appropriate sign and whose amplitude is between the threshold vertical select-field values for the two bead populations would direct large beads along Path 2 and small beads along Path 1. This capability is demonstrated in Fig. 5(b–i). Figure 5(b) shows the approach of a large and small bead to a junction. As the large bead enters the junction (Fig. 5(c)), it triggers ROI1 (rectangle) and ${H}_{z}=$ 35 Oe is applied. Because this vertical field is larger than the threshold vertical select-field for the large bead, the bead continues along Path 2 (Fig. 5(d,e)). However, when the small bead enters the junction and triggers the same ${H}_{z}=$35 Oe vertical field (Fig. 5(f)), it continues along Path 1 (Fig. 5(g–i)). In this case, the amplitude of the triggered vertical field is smaller than that of the threshold vertical select-field for a small bead. Thus, because directed bead motion depends on bead-DW interaction, the same stimulus can result in different responses and we are able to demonstrate a simple mechanism for the sorting of a mixed two-bead population. This behavior is repeatable and reliable, as demonstrated by the staticstics in the select field for the two bead types shown in Fig. 5(a).

## Discussion

The goal of lab-on-chip systems is the fast, accurate, and automatic manipulation of biological species. This requires compatibility among the various mechanisms that are necessary to provide device functionality. Previously, we reported on a proposed scheme for analyte detection in which changes in the hydrodynamic radius of a SPM bead, due to analyte hybridization with the functionalized bead, could be detected via a shift in the magneto-mechanical resonance of a bead driven by an oscillating DW^11, 12^. The mechanism for directed transport reported here enables the routing and sorting of beads after such identification and is simultaneously compatible with our previous architecture, which we have shown enables transport speeds approaching 1 mm/s^22^. Although we have demonstrated the sorting of a 2-bead population, in principle, populations with more than two species could be sorted by a sequence of junctions so long as the select fields for the bead species are sufficiently distinct from one another.

Furthermore, more complex routing networks with enhanced sorting functionality can easily be envisioned with simple track modification. We have shown in the present manuscript the behavior of beads at circular nodes with three junctions. However, the number of junction branch points need not be limited to three. With the manipulation technique described, an arbitrarily large number of branch points can be defined off a circular node, and is only limited by the space required for each branching track. With more available paths comes an increase in potential device functionality.

Finally, unlike other schemes in which filtering behavior is predetermined by the nature of the device, the behavior in this system is dynamic. That is, though the select-field for a bead will be a function of its interaction with the track, whether a bead goes along e.g. Path 1 or 2 is not fixed, but rather controlled by an external stimulus. This allows for dynamic filtering despite the fixed track pattern.

## Conclusions

This essential new capability of actively routing beads along specific routes in a complex nanotrack network adds a key building block for realizing a complete magnetic lab-on-a-chip system. In conjunction with the capability for high-speed transport^22, 23^ and resonant detection^11, 12^ of individual beads, the bead-DW system has exciting and promising application in future lab-on-a-chip technologies.

## Methods

### Sample preparation

Arrays of Ni_80_Fe_20_ (40 nm)/Pt (2 nm) branched curvilinear tracks on Si(100) wafer were prepared by electron beam lithography, dc sputtering, and liftoff. Each track was 800 nm wide and composed of linked semi-circular segments with a 20 *μ*m outer diameter. After patterning, the wafers were coated with a 70 nm thick rf-sputtered protective SiO_2_ overlayer. Experiments were performed using commercial Dynabeads M-270 Carboxylic Acid superparamagnetic beads (2.8 μm diameter) from Life Technologies, and COMPEL Magnetic, COOH modified (UMC3N/11086) beads (5.8 μm diameter) from Bangs Laboratories. Beads were suspended in water at a concentration of ~2 $\times$ 10^4^ beads/mL.

### Data availability

The datasets generated during and/or analyzed during the current study are available from the corresponding author on reasonable request.

## Electronic supplementary material

Supplemental Video 1
